# Frequency of Systemic Lupus Erythematosus Was Decreasing Among Hospitalized Patients From 2013 to 2017 in a National Database in China

**DOI:** 10.3389/fmed.2021.648727

**Published:** 2021-04-06

**Authors:** Ying Tan, Feng Yu, Jianyan Long, Lanxia Gan, Haibo Wang, Luxia Zhang, Minghui Zhao

**Affiliations:** ^1^Renal Division, Department of Medicine, Peking University First Hospital, Beijing, China; ^2^Peking University Institute of Nephrology, Beijing, China; ^3^Key Laboratory of Renal Disease, Ministry of Health of China, Beijing, China; ^4^Key Laboratory of CKD Prevention and Treatment, Ministry of Education of China, Beijing, China; ^5^Research Units of Diagnosis and Treatment of Immune-mediated Kidney Diseases, Chinese Academy of Medical Sciences, Beijing, China; ^6^Department of Nephrology, Peking University International Hospital, Beijing, China; ^7^China Standard Medical Information Research Center, Shenzhen, China; ^8^Center for Data Science in Health and Medicine, Peking University, Beijing, China; ^9^Peking-Tsinghua Center for Life Sciences, Beijing, China

**Keywords:** systemic lupus erythaematosus, lupus nephritis, frequency, hospitalized population, mortality

## Abstract

**Backgrounds:** Limited data was reported for the frequency of SLE in China. The aim of this study was to investigate the frequency, geographical, and ethnic distributions of hospitalized SLE patients with data from the Hospital Quality Monitoring System (HQMS) in China.

**Methods:** Hospitalized patients were investigated from a national inpatient database covering 46.0% of tertiary hospitals in China from 2013 to 2017. Data regarding the diagnosis of SLE were extracted based on ICD-10 codes. We collected and analyzed data from the front page of the records of inpatients, including frequency, demographic characteristics, and geographic distributions of SLE.

**Results:** Among 158.3 million inpatients attended during the study period, 0.31% (491, 225) were diagnosed with SLE. The frequency of SLE decreased during the study period (from 0.30% in 2013 to 0.27% in 2017). The frequency of SLE increased with latitude (0.21% in northern China and 0.39% in southern China in 2017). Hospitalizations mostly occurred in winter (31.24%). The Li population had the highest frequency of patients with SLE (0.76%). The all-cause in-hospital mortality rate of SLE decreased from 0.74% (255/34,746) in 2013 to 0.54% (295/54,168) in 2017. The percentage of SLE patients with infections increased from 3.14% in 2013 to 4.72% in 2017. The percentage of SLE patients with tumors and thrombosis also increased slightly from 0.85 and 1.43% in 2013 to 1.27 and 2.45% in 2017, respectively.

**Conclusion:** This study provided epidemiological information of SLE in hospitalized patients in China for the first time. An ethnic and spatial clustering trend of SLE was observed.

## Introduction

Systemic lupus erythaematosus (SLE) is a systemic autoimmune disease that can affect multiple systems, including the kidneys, brain, haematologic system, and so on (1). Tremendous improvements in the diagnosis and medical care of SLE have resulted in an increase in the 5-year survival rate to over 90% and the 15–20-year survival rate up to about 80% since the 1950s, which include earlier diagnosis, renal replacement, dialysis, and medication (2). However, SLE still has a significant impact on morbidity, mortality, and quality of life, which results in heavy burden of society and the patients themselves, such as the insurance cost and productivity loss (3). The estimated prevalence of SLE might be useful in health care planning by increasing our understanding of the burden of disease.

The epidemiology of SLE has been studied worldwide in the past decades. The prevalence rate varied from 4.8 to 91 per 100,000 people across sexes, age groups, geographical regions, and ethnic backgrounds (4). The incidence and prevalence rates in people of African or Asian backgrounds were ~2–3-times higher than those in white populations, and higher mortality risks have been observed among the black and Hispanic populations than among white (or majority) populations (5). Previously, a large population-based epidemiological study indicated that the incidence of SLE in UK, southern USA, southern Sweden, and South Korea decreased and the prevalence increased (6–8).

The prevalence of SLE and lupus nephritis in China is not yet known. The estimated prevalence available have been based on populations in small geographic areas, such as in rural areas of Anhui Province (9). Any mechanical extrapolation of estimated prevalence of SLE obtained from other populations to local conditions might be misleading due to differences in the distribution of environmental and genetic risk factors.

The aim of this study was to estimate the nationwide frequency of SLE based on a national registration database with comprehensive geographic information inpatients.

## Materials and Methods

### Study Population

The study population comprised 158,335,283 inpatients from 1,064 tertiary hospitals from Jan 1st 2013 to Dec 31st 2017, covering 46.0% of tertiary hospitals in 31 provinces in China.

The inpatients database was obtained from the Hospital Quality Monitoring System (HQMS), a standardized registration dataset of the electronic discharge records for inpatients of tertiary hospitals around China. Almost all the tertiary hospitals submitted electronic inpatients discharge records to HQMS automatically, leading by the Bureau of Medical Administration and Medical Service Supervision and National Health and Family Planning Commission of the People's Republic of China since January 1st, 2013. General data, diagnosis, and expenditures were extracted from the “front page” of inpatient's medical records.

Physicians were responsible for filling in the data on the front page, and the diagnoses were coded based on the International Classification of Diseases-10 (ICD-10) coding system by certified professional medical coders at each participating hospital. Data quality was controlled at the time of data submission to automatically assure completeness, consistency, and accuracy.

For patients with more than one admission, only the first admission was included for analysis. A total of 491,225 patients were diagnosed as SLE and 252,074 patients were identified for the analysis from January 1st, 2013 to December 31st, 2017. The identification number and telephone numbers were used to define the residency. The health insurance type was used to discover the urban/rural residency. Basic medical insurance or free medical insurance indicate urban residency, while new rural cooperative medical care for rural residency. The ethics committee of Peking University First Hospital approved this study (2015[928]).

### Patients and Public Involvement

No patient or public were involved in the design, conduct, reporting, or dissemination plans of our research.

### Definition of SLE

The ICD-10 disease codes are used to identify SLE patients in tertiary hospitals in China according to the HQMS database, including the Beijing version 4.0, the national standard version 1.0, and the national clinical version 1.0 and the national clinical version 1.1 (Appendix 1). Patients with lupus vulgaris, discoid lupus erythaematosus of eye lid, discoid lupus erythaematosus, subacute cutaneous lupus erythaematosus, lupus erythaematosus NOS, panniculitis, erythaematosus profundus, fetus lupus erythaematosus, scrofuloderma, sycosis barbae, or drug-induced systemic lupus erythaematosus and fetuses or newborns affected by maternal systemic lupus erythaematosus were excluded (Appendix 2).

### Demographic Data and Other Covariates

Information on age, sex, ethnicity, occupation, residence, and health insurance was extracted from the front page of health records and included in the analysis. Outcome data on expenditures, duration, and in-hospital mortality were collected, too. The survival status of each patient was verified based on discharge status combined with information from autopsy records.

### Statistical Analysis

The frequency and absolute number of SLE patients were reported. Patients with SLE were stratified by age, sex, geographic region, and residency. Continuous data are presented as the mean ± standard deviation or as the median (inter-quartile range). Categorical variables are presented as proportions with 95% confidence intervals (CIs). All analysis were performed using SAS 9.1 (SAS Institute Inc., Cary, NC, United States).

## Results

### The Decreasing Frequency of SLE Among Inpatients From 2013 to 2017

**Figure 1:**
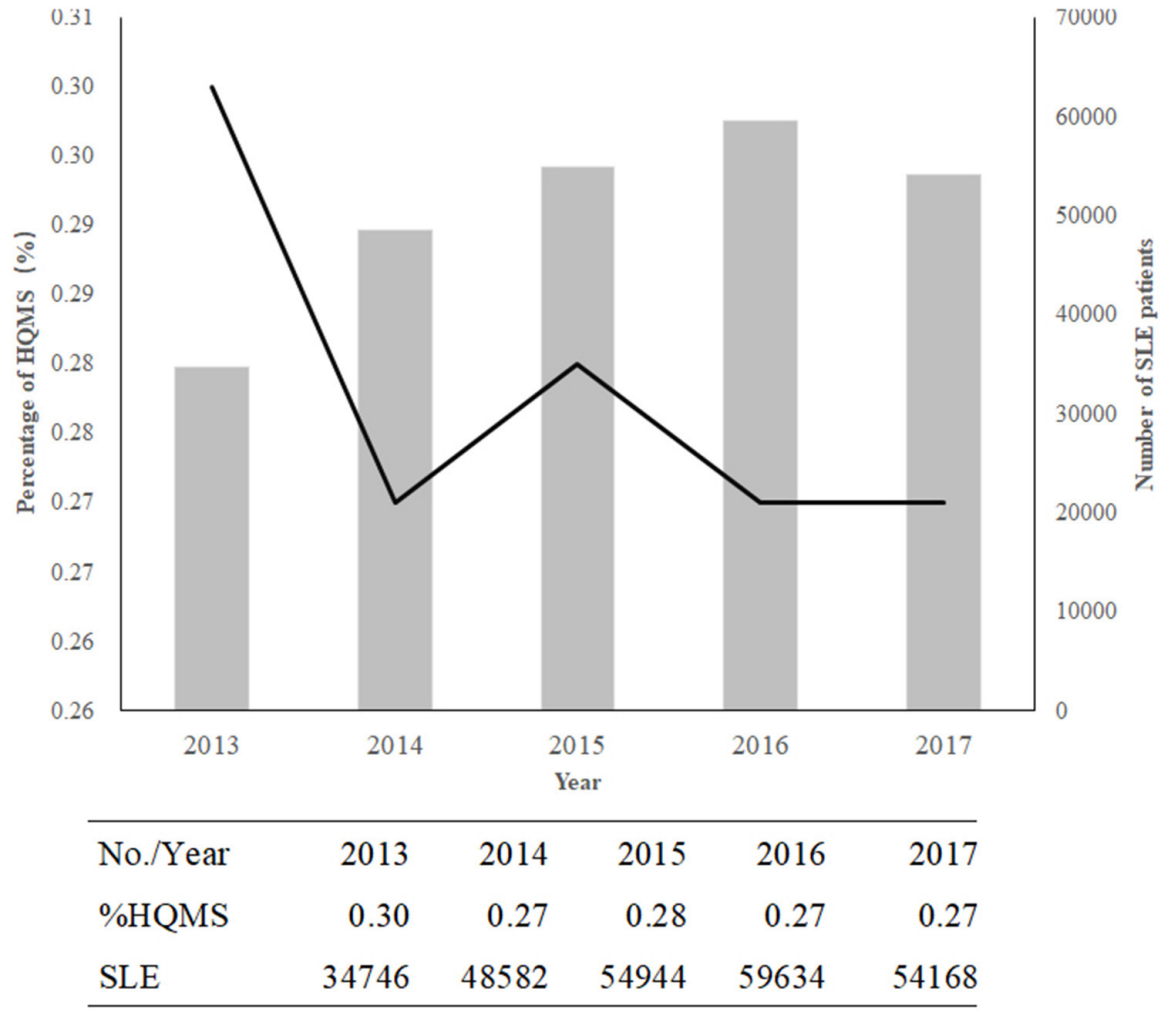
The decreasing trend in SLE in China from 2013 to 2017. The columns indicate the numbers of patients with SLE; the line shows the percentage of SLE or lupus in HQMS.

The frequency of SLE among inpatients decreased from 0.30% in 2013 to 0.27% in 2017, with a changing spectrum (Figure 1). However, the number of patients with SLE increased from 34,746 to 54168 (Figure 1). Hospitalizations were mostly observed in winter (31.24%).

### Percentage of SLE Stratified by Sex and Age Group

The percentage of SLE in HQMS was highest in age group 19–24 years during 2013–2017. SLE predominantly affected women (F/M 8-9:1). After stratification by sex, there were two peaks of age groups for the females (19–24 years and 40–44 years) and one for males (19–29 years) for the percentage of SLE in HQMS in 2017 (Supplementary Figure 1). The number of SLE patients was highest in the 25–29 years age group during 2013–2017.

### The Ethnic Distribution of SLE in 2017

**Figure 2:**
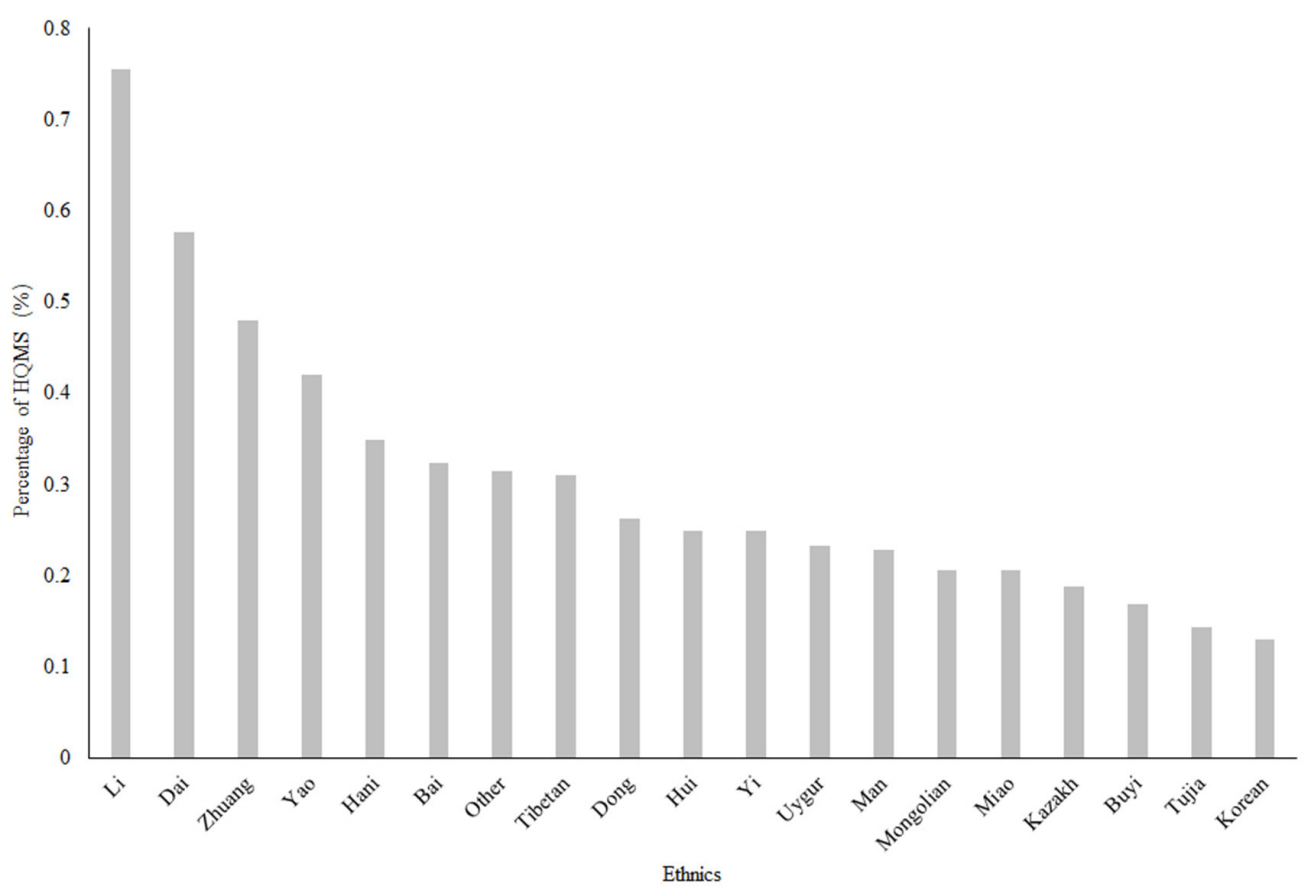
The ethnic distribution of SLE in China in 2017. The Li, Dai and Zhuang minorities had the higher frequencies of SLE, with frequencies of 0.755, 0.576, and 0.48%, respectively. HQMS, Hospital Quality Monitoring System Note: Information on races was missing for 11,648 (4.62%) of all 5-year SLE patients included.

The Li, Dai and Zhuang ethnic minorities had the highest frequencies of SLE, with frequencies of 0.755, 0.576, and 0.48%, respectively (Figure 2). The Li, Dai and Zhuang populations are mostly distributed in southern China and southwestern China.

### The Geographic Distribution of SLE

**Figure 3:**
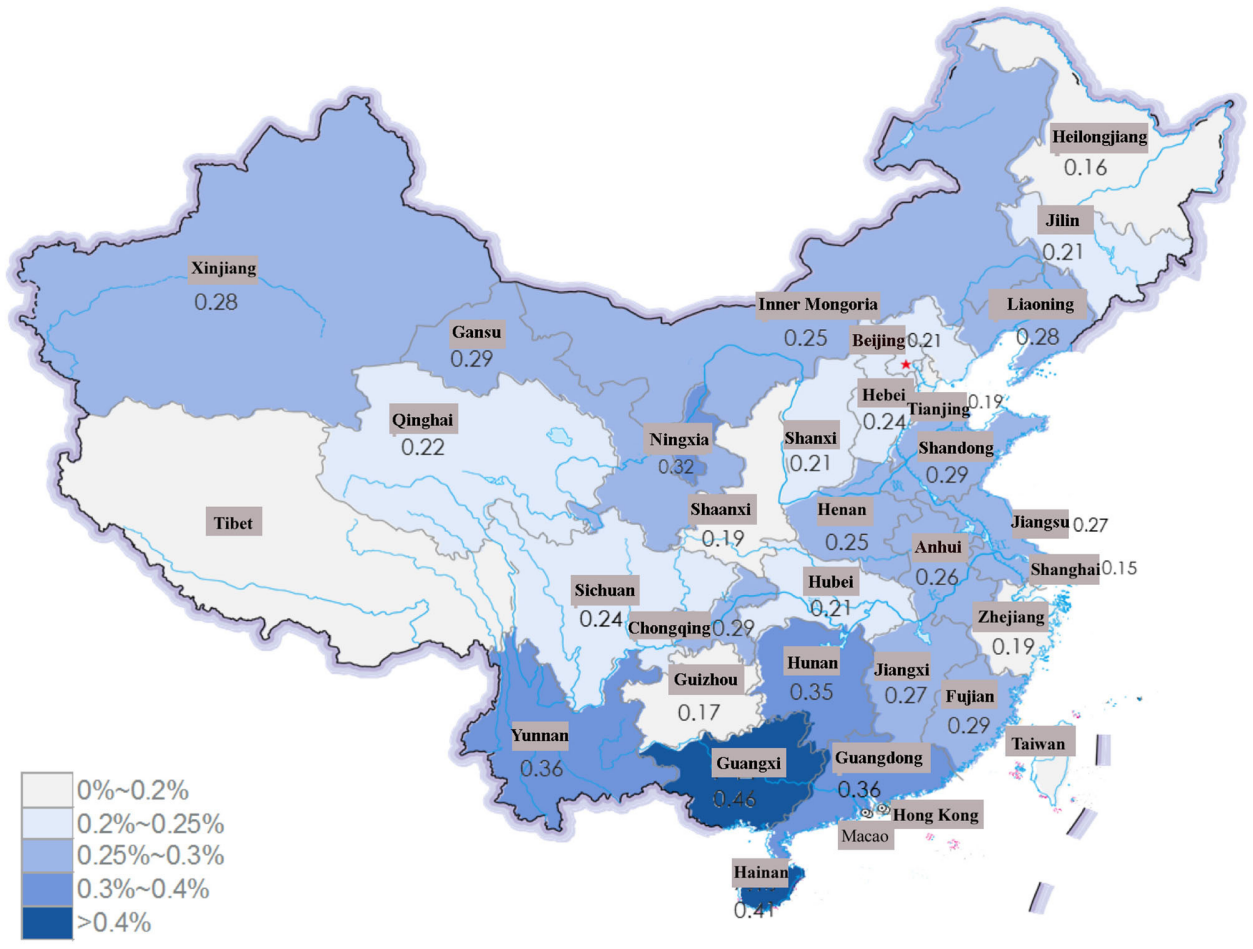
The frequency of SLE among all the patients in HQMS in province of China in 2017. The frequency of SLE was much higher in Guangxi Province, Hainan Province and Guangdong Province (0.46, 0.41, and 0.36%, respectively).

SLE patients were concentrated in southern China, and the frequency of SLE among inpatients showed a decreasing trend (Supplementary Figure 2). Among the 31 provinces included, the frequency of SLE was higher in Guangxi Province, Hainan Province and Guangdong Province (0.46, 0.41, and 0.36%, respectively) in 2017 (Figure 3). In urban areas, the frequency of SLE patients increased during the study period (from 57.99% in 2013 to 63.59% in 2017), while in rural areas, the frequency of SLE decreased from 42.01% in 2013 to 36.41% in 2017.

### Decreasing In-Hospital Mortality Rate of SLE in Different Age Groups

The all-cause in-hospital mortality of SLE decreased from 0.74% (255/34,746) in 2013 to 0.54% (295/54,168) in 2017. Patients in different age groups showed significantly different mortality rates (*P* < 0.001). The 25–29-year group showed the highest mortality rate among all age groups (Supplementary Figure 3). Patients with heart involvement showed the highest mortality among all other organ involvements.

### Characteristics of SLE With Different Organ Involvement

Demographic characteristics of SLE patients with different organ involvement admitted in 2017 were summarized (Table 1). Lupus nephritis was the most common complication of SLE, while heart involvement was the least common from 2013 to 2017 (Supplementary Figure 4).

**Table 1:** Selected characteristics of SLE patients with different organ involvements in 2017.

	**SLE**	**Lupus nephritis**	**Lung involvement**	**Hematological involvement**	**Gastrointestinal involvement**	**Neurologic involvement**	**Heart involvement**
Number	34,746	13,876	7,032	5,388	6,612	3,316	3,112
Age (years)	41.08 ± 15.88	39.27 ± 15.44	45.13 ± 16.56	40.61 ± 16.05	44.33 ± 15.58	47.10 ± 17.21	48.53 ± 17.71
Female:Male (ratio)	7.98:1	6.50:1	6.21:1	8.52:1	6.59:1	6.30:1	5:38:1
Occupation No. (%)^*^							
Clerk	4,669 (9.44)	1,755 (8.63)	1,010 (7.47)	959 (8.66)	1,181 (9.09)	428 (7.11)	416 (6.77)
Professional	1,147 (2.32)	481 (2.36)	281 (2.08)	273 (2.46)	291 (2.24)	121 (2.01)	108 (1.76)
Worker	2,413 (4.88)	966 (4.75)	601 (4.45)	514 (4.64)	668 (5.14)	263 (4.37)	292 (4.75)
Farmer	9,810 (19.83)	4,278 (21.03)	3,211 (23.75)	2,452 (22.13)	2,880 (22.17)	1,201 (19.95)	1,278 (20.79)
Student	2,678 (5.41)	1,314 (6.46)	535 (3.96)	658 (5.94)	512 (3.94)	267 (4.43)	221 (3.60)
Retired	3,480 (7.03)	2,468 (12.13)	1,486 (10.99)	1,365 (12.32)	1,515 (11.66)	694 (11.53)	658 (10.71)
Unemployed	5,857 (11.84)	1,151 (5.66)	1,248 (9.23)	676 (6.10)	1,188 (9.15)	828 (13.75)	864 (14.06)
Other	19,426 (39.26)	7,928 (38.98)	5,148 (38.08)	1,296 (11.70)	4,754 (36.6)	2,219 (36.85)	2,309 (37.57)
Rural/urban^*^ No. (%)							
Urban	23,465 (63.59)	9,250 (61.33)	6,361 (61.97)	5,023 (61.47)	6,451 (65.43)	3,229 (68.60)	3,213 (66.07)
Rural	13,434 (36.41)	5,832 (38.67)	3,904 (38.03)	3,149 (38.53)	3,409 (34.57)	1,478 (31.40)	1,650 (33.93)
Medical insurance No. (%)							
Basic medical insurance	23,465 (43.32)	9,250 (41.50)	6,361 (43.18)	5,023 (41.31)	6,451 (45.27)	3,229 (48.78)	3,213 (47.00)
New rural CMC	13,434 (24.80)	5,832 (26.17)	3,904 (26.50)	3,149 (25.90)	3,409 (23.92)	1,478 (22.33)	1,650 (24.13)
Other insurance	2,214 (4.09)	1,066 (4.78)	655 (4.45)	592 (4.87)	681 (4.78)	328 (4.96)	334 (4.89)
Un-insurance	8,926 (16.48)	3,613 (16.21)	2,281 (15.48)	2,048 (16.84)	2,143 (15.04)	895 (13.52)	933 (13.65)
Infection (%)	4,633 (8.55)	2,223 (9.97)	3,532 (23.98)	1,558 (12.81)	1,671 (11.73)	712 (10.76)	980 (14.33)
Tumors (%)	1,248 (2.30)	275 (1.23)	356 (2.42)	189 (1.55)	341 (2.39)	150 (2.27)	132 (1.93)
Thrombosis (%)	2,399 (4.43)	909 (4.08)	912 (6.19)	652 (5.36)	716 (5.02)	1,808 (27.32)	596 (8.72)
Costs^*^ (1000RMB), Median (Q1-Q3)	8,862 (5,224–14,949)	9,576 (5,264–16,589)	12,460 (7,674–22,259)	11,947 (7,126–21,087)	10,569 (6,503–17,816)	11,324 (7,079–20,221)	12,654(7,596–23,819)
Length-of-stay^*^(days), Median (Q1-Q3)	9 (6–14)	10 (6–16)	12 (8–18)	12 (7–18)	11 (7–16)	11 (7–17)	11 (7–17)
In-hospital mortality (%)	295 (0.54)	158 (0.71)	215 (1.46)	127 (1.04)	116 (0.81)	101 (1.53)	153 (2.24)

*Continuous variables are presented as the mean ± standard deviation or as the mean (inter-quartile range) for age and cost; categorical variables are presented as percentages with 95% confidence intervals*.

* *Area total of 0.03–0.13% of age, 7.80–10.04% of occupation, 29.9–34.03% of rural/urban, 10.00–11.58% of costs, and 0.14–0.46% of stay-length data were missing*.

### The Sex Ratio of SLE With Different Organ Involvement

A female predominance was found in regard to different organ involvements among in-hospital SLE patients. The sex ratio (F/M) was higher in the group with hematological involvement than in patient groups with other organ involvements from 2013 to 2017 (Supplementary Figure 5).

### The Percentages of SLE Patients With Complications Among Different Organ Involvements

The percentage of SLE patients with infections increased from 3.14% in 2013 to 4.72% in 2017. The percentage of SLE patients with tumors and thrombosis also increased slightly from 0.85 and 1.43% in 2013 to 1.27 and 2.45% in 2017, respectively (Supplementary Figure 6A). Among SLE patients with different organ involvements, the percentages of patients with infection and thrombosis were the highest among patients with lung involvement (Supplementary Figures 6B,C). The percentages of patients with tumors were highest in the SLE patients with gastrointestinal involvement (Supplementary Figure 6D).

## Discussion

Using a large national registry database including 491,225 SLE patients, we first analyzed the changing frequency of SLE patient hospitalization with the most comprehensive geographic coverage in China from 2013 to 2017.

A decreasing frequency of SLE in inpatients from 2013 to 2017 was noticed in this study, although the total number of SLE patients increased during study period. Based on the data from the National Health and Family Planning Commission of the Peoples' Republic of China (10), we estimate that ~576,850 patients with SLE were treated in hospitals with the estimated prevalence 41.50 of per 100,000 people in China in 2017 (11), which places a heavy burden on the government and the public. The estimated prevalence was lower than the reports before since it was based on the in-hospitalized patients which might underestimate the prevalence (12). The change trend was similar to those observed in regions of other countries, such as in the United Kingdom (6), southern USA (7), and southern Sweden (13), among others (14).

Hospitalizations were mostly observed in winter in our study. Previous studies have shown that increased incidence of Epstein-Barr virus (EBV) infections was associated with lupus onset or flares during the winter (15). What's more, vitamin D insufficiency could be a risk factor for lupus flare in winter (16). These studies might provide an explanation of the hospitalizations of SLE which might because of disease onset or flare in winter.

The age peaks of males and females regarding SLE frequency in our study somewhat is similar to the data from South Korea but different from other studies of incidence and prevalence, such as studies from UK (8, 14). This may be due to differences in ethnicities and in data sources. The data was from in-hospitalized patients in our study and the studies from UK was from the General Practice Research Database (14).

The frequency of SLE increased with decreasing latitude (0.21% in northern China and 0.39% in southern China) in 2017. Incidence studies performed in southern Sweden suggested that there were significant geographic variations in the incidence of SLE (17): up to a 4-fold variation was observed by county. The association of frequency and latitude was also observed in Anti-neutrophil cytoplasmic autoantibody (ANCA)-associated vasculitis (AAV) in our previous study (18). The reason might be genetic variations and the ultraviolet radiation gradient due to different latitudes (19). Moreover, more patients were from urban rather than rural areas, with an increasing difference between the two areas observed in our study. This finding agreed with previous studies conducted in Greece, where the SLE was higher in urban residency (20, 21). Additionally, the incidence and prevalence of SLE were much higher in New York City than those in Jefferson County rural area (22). The difference between rural and urban areas indicates that the inhibited environment may be a risk factor of SLE, as previously reported in a large population-based study of the prevalence of SLE in rural regions of Anhui Province (9).

The Chinese Li, Dai and Zhuang populations had much higher frequency of patients with SLE in our study, with a frequency nearly 2–3 fold higher than the national average and high distribution in southwestern and southern China. The Li population mainly lives in Hainan Province, and the Dai population mainly lives in Yunnan Province, which might also contribute to the relatively high frequency of patients with SLE in these provinces compared to that in provinces at the same latitude, like those in eastern China. Genome-wide association studies (GWAS) have shown that a total of over 60 loci are related to SLE, including HLADR, TLR5, and TLR9 (23). The most common DR-specific locus is HLA-DR2 in the Dai population with a frequency much higher than that in other Chinese populations (24). Phylogenic studies have revealed that the Chinese Li and Dai populations were distinct from the Han population (25). Previous studies have shown that the TLR9-MyD88-TRAF6-IRF5 signaling pathway has a certain relationship with the development of SLE (26, 27). Wen et al. showed that TRAF6 rs5030472 and IRF5 rs2004640 gene polymorphisms may be related to SLE susceptibility in the Guangxi Zhuang population, while the TLR9 rs352140 gene polymorphism may be associated with SLE susceptibility in the Guangxi Han population, which suggested that ethnic differences existed (28). Further genetic studies might be able to reveal the genetic variants related to SLE in this race, which might provide a promising opportunity to further explore the pathogenesis of SLE in China.

The all-cause in-hospital mortality of SLE was decreasing in our study from 2013 to 2017. It was encouraging that SLE mortality have decreased during the study period, and the trend was similar in other countries, which might have resulted from the improvement of therapies and early diagnosis (29, 30). However, the mortality of SLE remained higher compared with that in the general population. Moreover, the young patient group (25–29 years) still had the highest mortality rate among other groups in our study. As in the US, SLE was the leading causes of death among young women from 2000 to 2015 (31). The high mortality of young people makes it an important public health issue since both demographic and geographic variables contribute to the development (32).

Our study found that the percentage of SLE patients with infections increased from 3.14% in 2013 to 4.72% in 2017. Infections are associated with the molecular mimicry of autoantigens, abnormal production of autoantibodies, lack of response from immune system and the development of SLE, such as EBV infection (33). However, recent studies have indicated that some pathogens (such as malaria parasites, hepatitis B virus, *Toxoplasma gondii*, and *Helicobacter pylori*) might be protective against SLE (34). Thus, the increase in infections could be associated with the complications of steroids and immunosuppressants or the causative or protective effects in regard to SLE. The different types of infectious agents should be clarified and need further investigation in the future.

The percentage of SLE patients with tumors was slightly elevated in our study. This finding was in accordance with a group of previous studies that showed that SLE patients had an increased risk of development of cancer (35, 36). A nationwide population-based study from Taiwan found that pediatric SLE patients were more susceptible to malignancy than the non-SLE children based on a sample of 904 SLE pediatric patients followed for 6 years in Taiwan's registry (37). However, the extent of this increased risk has yet to be determined in larger studies before setting up special screening guidelines for tumors in SLE patients.

The frequency of thrombosis in SLE patients was increased slightly in our study from 2013 to 2017. Patients with SLE were reported to have an increased risk of thrombosis, with a thrombotic history documented in between 9 and 30% of patients (38–40). Previous studies indicated that thrombosis could be a risk factor for the morbidity and mortality in patients with SLE. Patients are more susceptible with older age, smoking, immunomodulating medication, genetic mutations, and the presence of aPL antibodies (41, 42).

Some limitations existed in our study. First, the study included data from only inpatients in China, which makes the estimation of the total people at risk in China improbable; thus, the prevalence or incidence of SLE could not be clearly defined. However, the inpatient's information facilitates to show the socio-economic burdens of SLE. Second, all diagnosis were extracted but without detailed information on clinical manifestations, additional examinations or medications, which makes it impossible for further analysis.

## Conclusion

In conclusion, this study provided epidemiological information on SLE from hospitalized patients in China for the first time. An ethnic and spatial clustering trend of SLE was observed.

## Data Availability Statement

The datasets used or analyzed during the current study are available from the corresponding author on reasonable request.

## Ethics Statement

The studies involving human participants were reviewed and approved by the ethics committee of Peking University First Hospital approved this study (2015[928]). Written informed consent to participate in this study was provided by the participants' legal guardian/next of kin.

## Author Contributions

YT analyzed, interpreted the patient data, and a major contributor in writing the manuscript. JL, LG, and HW analyzed the raw data. FY, LZ, and MZ designed the study and substantively revised it. All authors contributed to the article and approved the submitted version.

## Conflict of Interest

The authors declare that the research was conducted in the absence of any commercial or financial relationships that could be construed as a potential conflict of interest.
