# Supplementary figures and images for: Frequency of Systemic Lupus Erythematosus Was Decreasing Among Hospitalized Patients From 2013 to 2017 in a National Database in China
Source: Front Med (Lausanne). 2021 Apr 6;8:648727. doi: 10.3389/fmed.2021.648727 (PMC8056078; doi:10.3389/fmed.2021.648727)

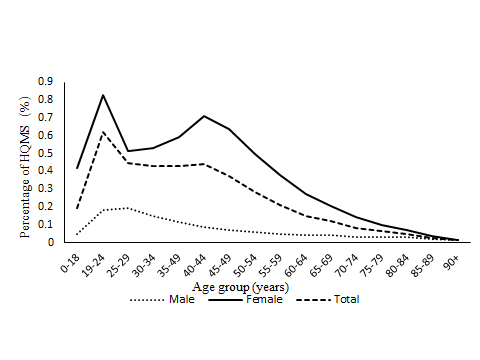

Supplement: Supplementary file 1 [file Image_1.JPEG]

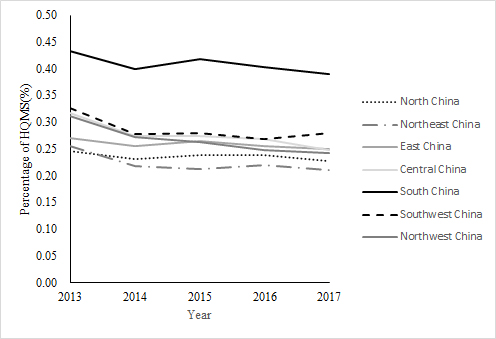

Supplement: Supplementary file 2 [file Image_2.JPEG]

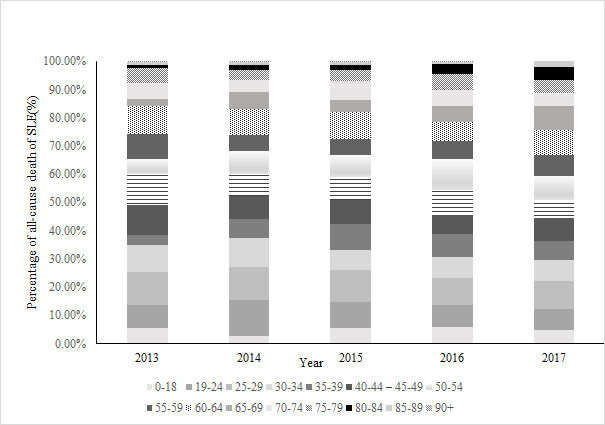

Supplement: Supplementary file 3 [file Image_3.JPEG]

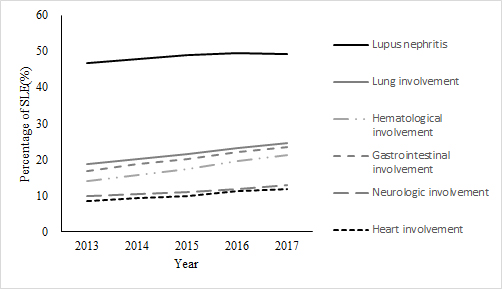

Supplement: Supplementary file 4 [file Image_4.JPEG]

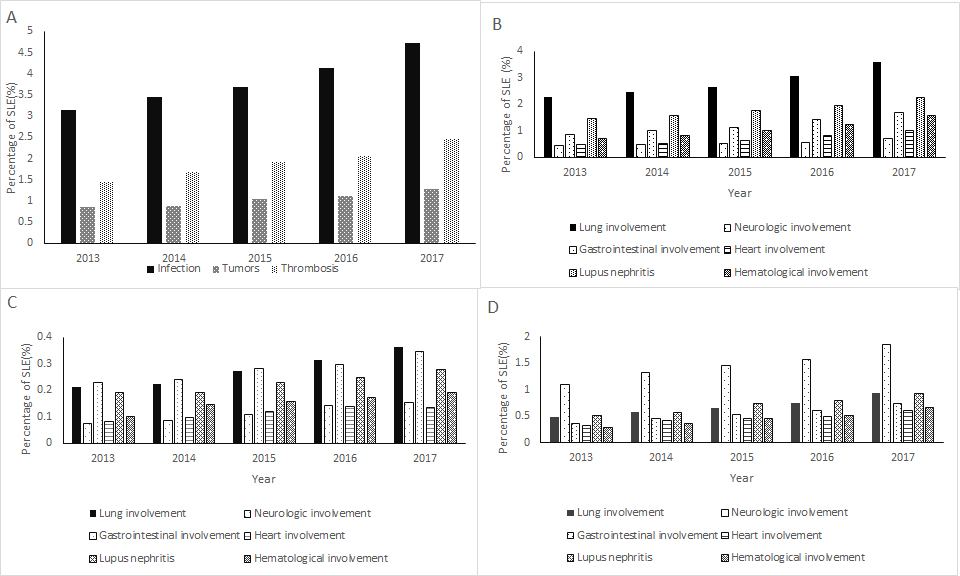

Supplement: Supplementary file 5 [file Image_5.JPEG]
