# Supplementary material for: Frequency of Systemic Lupus Erythematosus Was Decreasing Among Hospitalized Patients From 2013 to 2017 in a National Database in China
Source: Front Med (Lausanne). 2021 Apr 6;8:648727. doi: 10.3389/fmed.2021.648727 (PMC8056078; doi:10.3389/fmed.2021.648727)
Supplement: Supplementary file 7 [file Data_Sheet_1.docx]

**Supplementary figure legends**

**Supplementary Fig. 1. Frequency of SLE stratified by sex and age group in 2017**

**Supplementary Fig. 2. The fluctuation of percentage of SLE in seven geographic regions of China from 2013-2017**. SLE was more prevalent in South China. The decreasing trend was almost the same among different geographic regions.

**Supplementary Fig. 3. The percentage of all-cause death in each age group of SLE in 2017.**

The 25-29yrs group showed the highest mortality rate among all the age groups

**Supplementary Fig. 4. The percentage of SLE with different organ involvements from 2013-2017**.

Lupus nephritis was the most common complication of SLE, while heart involvement was the least common from 2013-2017.

**Supplementary Fig. 5. The sex ration of SLE patients with different organ involvement.**

The sex ratio (F:M) was higher in the hematological involvement than other organ involvement.

**Supplementary Fig. 6. The percentage of SLE patients with complications among different organ involvement.**

A, the percentage of SLE patients with infection, thrombosis and tumors; B the percentage of SLE patients with infection in different organ involvement, C the percentage of SLE patients with thrombosis in different organ involvement, D the percentage of SLE patients with tumors in different organ involvement.
