# Supplementary material for: Frequency of Systemic Lupus Erythematosus Was Decreasing Among Hospitalized Patients From 2013 to 2017 in a National Database in China
Source: Front Med (Lausanne). 2021 Apr 6;8:648727. doi: 10.3389/fmed.2021.648727 (PMC8056078; doi:10.3389/fmed.2021.648727)
Supplement: Supplementary file 8 [file Table_1.docx]

**Appendix 1 The International Classification of Diseases-10 coding of SLE**

| **Diseases** | **National Standard Version1.0** | **Beijing Version 4.0** | **National Clinical Version 1.1** |
| --- | --- | --- | --- |
| SLE | L93.001, M32.100, M32.800, M32.901, O99.811 | L93.001, M32.900,  M32.901, O99.807 | L93.001  M32.100  M32.901,  O99.811,  M32.900,  M32.800, |
| Lung involvement of SLE | \| M32.103+J99.1,  M32.115, \| \| --- \| | M32.107+J99.1, M32.104+J99.1, M32.111+K67.8 | M32.103+J99.1*  M32.115+K67.8* |
| Neurologic involvement of SLE | M32.106+G63.5, M32.107+G99.2, M32.113+H36.8, M32.114+G94.8,  M32.110+G73.7 | M32.108+G63.5, M32.110+G63.5, M32.109+G99.2, F06.921, M32.101+G05.8 | M32.106+G63.5* M32.107+G99.2*  M32.114+G94.8*，  M32.110+G73.7*,  M32.100x014+G73.7*,  F06.800x021  M32.113+H36.8* |
| Gastrointestinal involvement | M32.108+K77.8, M32.111+D77, M32.112+K93.8 | K71.502, K73.201, | M32.108+K77.8*,  K71.500x002  K73.200x001,  M32.112+K93.8*,  M32.100x018+K93.8* |
| Heart involvement of SLE | M32.105+I32.8, M32.103+I43.8, M32.109+I39.8, | M32.102+I32.8, M32.103+I41.8, M32.106+I39.8 | M32.105+I32.8*, M32.109+I39.8*,  M32.104+I43.8*，M32.100x006+I39.8* |
| Lupus nephritis | M32.101+N08.5, M32.102+N16.4, | M32.105+N08.5, M32.112+N08.5, M32.113+N16.4 | M32.101+N08.5*  M32.102+N16.4* |
| Hematological involvement of SLE | M32.111+D77 |  | M32.111+D77* |

Abbreviations: SLE systemic lupus erythematosus
