# Supplementary material for: Frequency of Systemic Lupus Erythematosus Was Decreasing Among Hospitalized Patients From 2013 to 2017 in a National Database in China
Source: Front Med (Lausanne). 2021 Apr 6;8:648727. doi: 10.3389/fmed.2021.648727 (PMC8056078; doi:10.3389/fmed.2021.648727)
Supplement: Supplementary file 9 [file Table_2.docx]

**Appendix 2 The International Classification of Diseases-10 coding of exclusion of SLE**

| **Diseases** | **National Standard Version1.0** | **Beijing Version 4.0** | **National Clinical Version 1.1** |
| --- | --- | --- | --- |
| Lupus vulgaris | A18.411 | A18.410 | A18.410 |
| Discoid lupus erythematosus of eye lid | H01.106 |  | H01.100x006 |
| Discoid lupus erythematosus | L93.002 | L93.000 | L93.000 |
| Subacute cutaneous lupus erythematosus | L93.101 | L93.100 | L93.100 |
| Lupus erythematosus NOS |  | L93.200 | L93.200x003  L93.200 |
| Panniculitis |  | L93.201 | L93.201 |
| Erythematosus profundus |  | L93.202 | L93.202 |
| Fetus or newborn affected by maternal systemic lupus erythematosus | P00.801 | P00.807 | P00.807 |
| Fetus lupus erythematosus |  | P00.814 | P00.814 |
| Fetus lupus erythematosus syndrome |  |  | P00.800x001 |
| Scrofuloderma | A18.401 | A18.409 | A18.409  A18.400x001 |
| Sycosis barbae |  | L73.801 | L73.801 |
| Drug-induced systemic lupus erythematosus | M32.001 | M32.000 | M32.000 |
| Lupus pernio |  |  | D86.300x002 |
